# Pushing the envelope

**DOI:** 10.7554/eLife.26397

**Published:** 2017-04-13

**Authors:** Julia H Wildschutte, John M Coffin

**Affiliations:** 1 1 Department of Biological Sciences, Bowling Green State University, Bowling Green, United States; 2 2 Department of Molecular Biology and Microbiology, Tufts University, Boston, United Statesjohn.coffin@tufts.edu

**Keywords:** endogenous retrovirus, hominid, paleovirology, receptors, virology, Human, Virus

## Abstract

Primates have co-opted a viral gene to produce an envelope protein that prevents infection by the HERV-T virus and likely contributed to the extinction of this virus.

**Related research article** Blanco-Melo D, Gifford RJ, Bieniasz PD. 2017. Co-option of an endogenous retrovirus envelope for host defense in hominid ancestors. *eLife* **6**:e22519. doi: 10.7554/eLife.22519

To counter the constant threat posed by viruses, vertebrate species have evolved a variety of antiviral mechanisms. In return, however, rapid mutation and turnover rates permit viruses to swiftly evolve to evade such mechanisms. This on-going ‘arms race’ between viruses and their hosts has had an important role in shaping the evolution of the species we observe today (tenOever, 2016).

Before a virus infects a cell, envelope proteins displayed on its surface must bind to a receptor located on the surface of the host cell. This receptor-envelope interaction is highly specific and, in turn, determines whether the virus is able to infect a particular cell type or species. In other words, in the absence of the receptor the virus loses its ability to infect the cell.

In a process termed ‘receptor interference’, an envelope protein from a previous viral infection can block cell receptors, preventing infection by a new virus – even an unrelated one – that also binds to that receptor (Figure 1). Remarkably, envelope proteins from such pre-infecting retroviruses can mediate receptor interference even if they have been extinct for millions of years (Boeke and Stoye, 1997). To date, clear examples of such interference have been limited to animal models, including chickens (Payne et al., 1971) and mice (Buller et al., 1987). Now, in eLife, Daniel Blanco-Melo, Robert Gifford and Paul Bieniasz of the Rockefeller University and MRC-University of Glasgow Centre for Virus Research report the first example of such interference in humans (Blanco-Melo et al., 2017).

**Figure 1.**
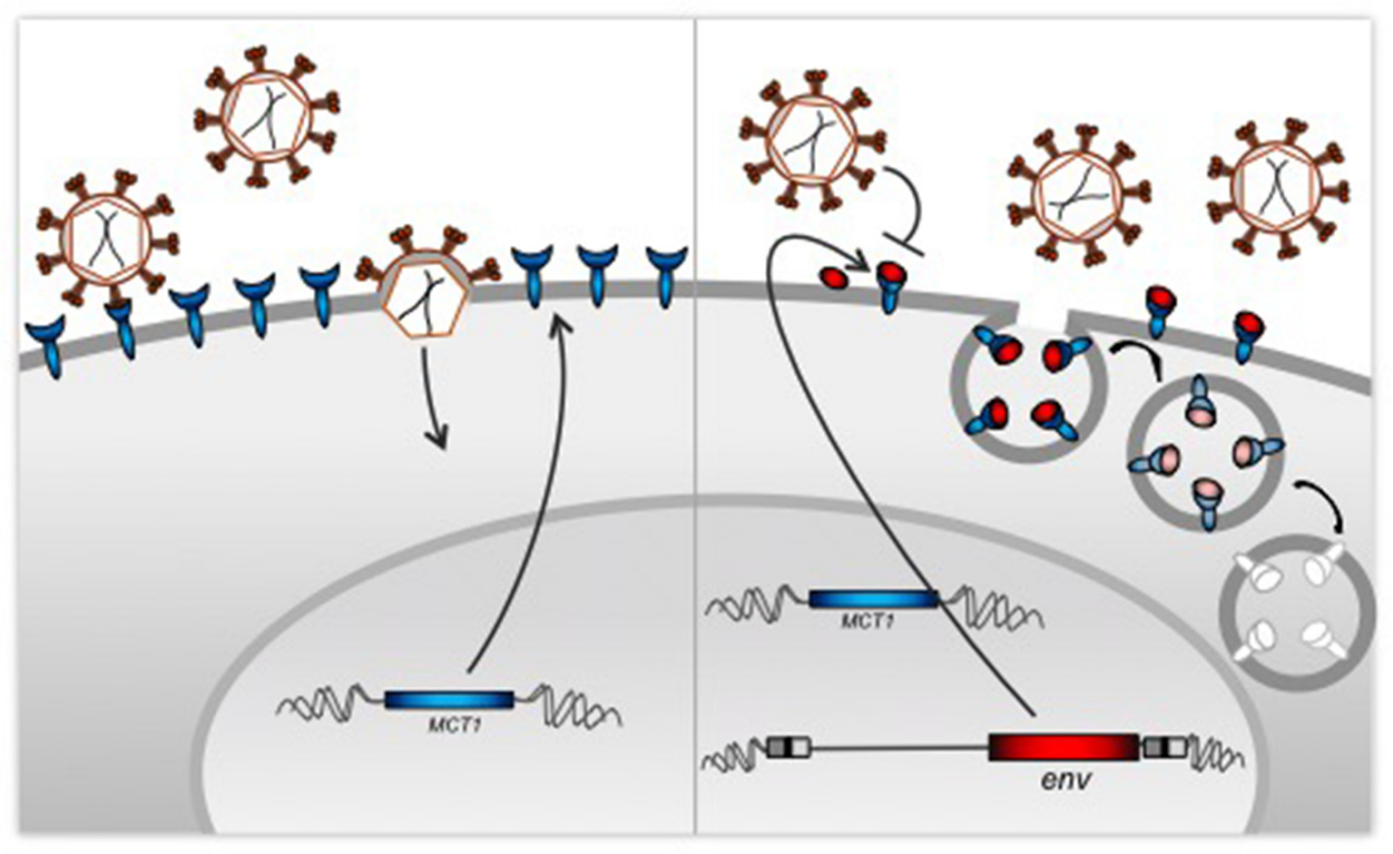
Co-option of a viral protein for receptor interference. Left: Cells expressing MCT1 (blue), which is the receptor for a retrovirus called HERV-T, are susceptible to infection from a virus that encodes a surface envelope protein produced by an ancestral form of HERV-T (red). Right: Our genome contains proviruses – copies of the DNA of ancient retroviruses, including HERV-T. Blanco-Melo et al. found that human cells can still produce envelope proteins from their copy of the *env* gene of the HERV-T provirus. These envelope proteins protect the cells from the resurrected virus by blocking the MCT1 receptors directly, or through the degradation of the resulting receptor-protein complex.

When a retrovirus infects a cell it integrates a DNA copy of its own genome (called a provirus) into the host cell’s genome. Because retroviruses occasionally infect germ line cells, the infection may lead to a provirus that is passed to offspring, and that can sometimes become fixed in the population. We refer to such proviruses as ‘endogenous retroviruses’. Over millions of years, large numbers of endogenous retroviruses have accumulated within vertebrate genomes, including humans, thus providing a ‘fossil record’ of previously circulating retroviruses that covers vast evolutionary scales.

While the majority of endogenous retrovirus lineages are ancient and now contain many mutations, recently formed examples tend to more closely resemble their infectious counterparts. This similarity means that, unless it is harmful, an endogenous retrovirus may retain the ability to produce functional RNA and protein products for long periods of time. Indeed, a few such endogenous retroviruses have been ‘co-opted’ to produce RNA or proteins that benefit the host (Ting et al., 1992; Mi et al., 2000; Stoye and Coffin, 2000).

The HERV-T lineage of endogenous retroviruses is an ancient member of a large group of retroviruses called gammaretroviruses (which includes leukemia viruses that affect cats and rodents). As detailed by Blanco-Melo et al., the HERV-T ancestor appears to have first invaded primate germlines about 43 to 32 million years ago, with the last invasion happening around 8 million years ago. Why did this lineage become extinct? Blanco-Melo et al. used an approach known as ‘paleovirology’ (Emerman and Malik, 2010) to begin to investigate this question.

By assessing the distribution of fossilized endogenous retroviruses among modern primate species, Blanco-Melo et al. identified a single HERV-T locus that appeared in the germline approximately 19 to 7 million years ago, whose envelope (*env*) gene, remarkably, has retained the ability to be translated. As other genes in the same provirus have been inactivated by mutations, this observation strongly suggests that this *env* gene has been selectively retained. However, its product cannot perform any ‘normal’ retroviral functions. Why, then, should this gene have been so clearly preserved throughout evolution?

Blanco-Melo et al. reconstructed an ancestral HERV-T sequence from the HERV-T proviruses found in the genomes of modern humans and other primates. A variety of cell lines could be infected by reconstructed viruses that encoded the ancestral *env* product, but those cells that had the version of *env* that is found in the host were resistant to infection. Blanco-Melo et al. then demonstrated that the product of the host-maintained *env* gene is able to block infection by viruses that encode the ancestral *env* gene by depleting the receptor they identified as MCT-1 (monocarboxylate transporter-1) from the surface of the host cell. The results imply that the HERV-T *env* gene in the host was co-opted and selected for antiviral protection through receptor interference.

The work from Blanco-Melo et al. highlights how endogenous retroviruses can act as raw material for potential use by the host in the host-virus arms race. This result raises the issue of the extent to which the co-option of endogenous retrovirus coding material for antiviral protection is widespread among vertebrates. Could such maintained *env* genes have contributed to the apparent extinction of other infectious retroviruses in the lineage leading to contemporary humans? We are eager to see whether the HERV-T picture represents a predictable pattern among endogenous retroviruses in other vertebrate lineages.
